# Cases of visual impairment caused by cerebral venous sinus occlusion-induced intracranial hypertension in the absence of headache

**DOI:** 10.1186/s12883-018-1156-7

**Published:** 2018-09-29

**Authors:** Tongtao Zhao, Gang Wang, Jiaman Dai, Yong Liu, Yi Wang, Shiying Li

**Affiliations:** 1 Department of Ophthalmology, Southwest Hospital, The Third Military Medical University (Army Medical University), Chongqing, China; 2 Aier Eye Hospital, Chongqing, China

**Keywords:** Cerebral venous sinus occlusion, Intracranial hypertension, Papilledema

## Abstract

**Background:**

Cerebral venous sinus thrombosis or stenosis (here collectively referred to as cerebral venous sinus occlusion, CVSO) can cause chronically-elevated intracranial pressure (ICP). Patients may have no neurological symptoms other than visual impairment, secondary to bilateral papilledema. Correctly recognizing these conditions, through proper ophthalmological examination and brain imaging, is very important to avoid delayed diagnosis and treatment.

**Case presentation:**

We report a case series of 3 patients with chronic CVSO, who were admitted to an ophthalmological department in Chongqing, China, from 2015 March to 2017 February. All patients presented with decreased vision and bilateral papilledema, but had no headache or other neurological symptoms. The visual fields of all patients were impaired. Flash visual evoked potentials (VEPs) in two patients showed essentially normal peak time of P2 wave, and pattern VEPs in one patient displayed decreased P100 amplitude in one eye, while a normal P100 wave in the other eye. In all patients, lumbar puncture (LP) revealed significantly elevated ICP. And magnetic resonance venography (MRV) demonstrated cerebral venous sinus abnormalities in every patient: one right sigmoid sinus thrombosis, one superior sagittal sinus thrombosis, and one right transverse sinus stenosis.

**Conclusions:**

CVSO can cause chronically-elevated ICP, leading to bilateral papilledema and visual impairment. A considerable amount of patients have no apparent neurological symptoms other than visual loss. Unlike other optic nerve lesions, such as neuritis or ischemic optic neuropathy, the optic disc edema in CVSO is usually bilateral, the flash or pattern VEP is often normal or only mildly affected, and patients are often not sensitive to steroid therapy. CVSO should be suspected in such patients when unenhanced brain imaging is normal. Further investigations, such as LP and contrast-enhanced imaging (MRV and digital subtraction angiography), should be performed to diagnose or exclude CVSO.

## Background

Cerebral venous sinus diseases, such as cerebral venous sinus thrombosis (CVST) and cerebral venous sinus stenosis (CVSS), are conditions affecting the intracranial venous drainage, causing either complete or partial cerebral venous sinus occlusion (CVSO) [1, 2]. This typically leads to elevated intracranial pressure (ICP), which can cause papilledema (usually bilateral) and visual impairment. When CVSO is acute (e.g., secondary to acute CVST), it typically causes severe headache and stroke-like neurological signs. However, when the onset is chronic, the symptoms of elevated ICP, like headache, are often absent, and the diagnosis may be delayed [3]. Here we report a case series of 3 patients with CVSO and elevated ICP, who had no apparent symptoms other than visual impairment, and firstly were admitted to ophthalmologists clinics.

## Case presentation

### Case 1

A 20 year-old man presented with a 1-month history of impaired vision, binocular horizontal diplopia and metamorphopsia. There was no history of headache, vomiting, fever, or trauma. He denied any history of hematological or neurological diseases, and was not on any medication. Notable in his past medical history was that he had undergone surgery for mastoiditis 8 years previously.

**Fig. 1:**
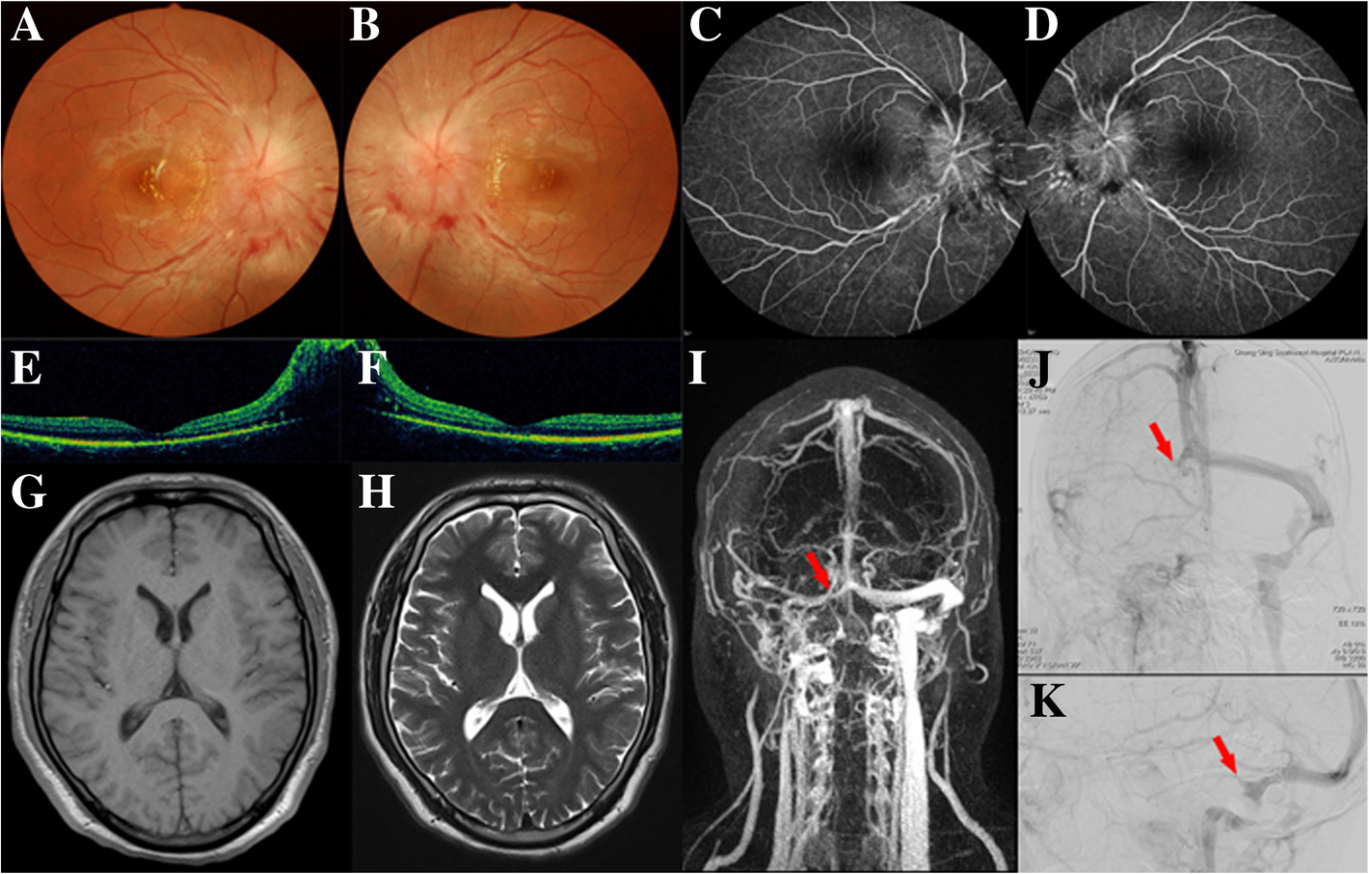
*Case 1, 20-year-old male patient with severe bilateral papilledema* **a, b,** Ophthalmoscopy images of right and left eye, respectively. **c, d,** FFA images, showing hyperfluorescence of optic discs bilaterally, without other abnormal leakage. **e f,** OCT images, showing relatively normal macular morphology in both eyes. **g, h,** Unenhanced T1- and T2-weighted MRI images, respectively, showing no abnormalities. **i,** MRV image, showing a poorly-visualized right sigmoid and transverse sinuses (*red arrow*). **j, k,** DSA images, showing a filling-defect (*red arrows*) in the right sigmoid and transverse sinuses

On presentation, the patient appeared in clear consciousness. Vital signs were stable, with blood pressure 121/82 mmHg, pulse 88 bpm and a body temperature of 37 °C. Best corrected visual acuity was 0.15 (Decimal Fraction) in both eyes. Ocular motilities of both eye were normal. Ophthalmoscopy revealed significant bilateral optic disc swelling with peri-papillary hemorrhages (Fig. 1a, b), but the eyes were otherwise normal. Fundus fluorescein angiography (FFA) showed hyperfluorescent leaking defects at the optic discs (Fig. 1c, d). Humphrey automated perimetry (HAP) revealed bilateral inferior arcuate scotomas (Fig. 4). Optical coherence tomography (OCT) showed bilateral papilledema, but the macular morphology was normal (Fig. 1e, f). Flash visual evoked potentials (FVEPs) showed normal peak time of the P2 wave (Fig. 4). The electroretinogram (ERG) also showed normal retinal function. Routine hematological and biochemical tests showed no significant abnormalities. Unenhanced brain and orbital magnetic resonance imaging (MRI) showed neither abnormal signals nor any signs of increased intracranial pressure, such as enlarged ventricles or mid-line shift, partially empty sella, flattening of the globe, or enlarged optic nerve sheaths (Fig. 1g, h). The patient was examined by neurologist, and no positive neurological signs were found. Considering the poor vision of both eyes, he was administrated with systemic steroids, but the visual acuity did not improve afterwards.

Given the patient’s manifestation and ophthalmological and systemic investigations, primary optic neuropathies, including optic neuritis and ischemic optic neuropathy, were basically ruled out. Specialized investigations for intracranial pathology were therefore performed. Magnetic resonance venography (MRV) showed a loss-of-signal void in the right sigmoid sinus (Fig. 1i). LP at the time showed an elevated cerebrospinal fluid (CSF) opening-pressure of over 40 cm H_2_O (normal, 18–20 cm H_2_O). CSF protein, glucose, and cell counts were all within normal limits. After neurological consultation, a digital subtraction angiography (DSA) was performed, which showed a filling-defect in the right sigmoid sinus (Fig. 1j, k). The patient was diagnosed with right sigmoid sinus thrombosis, and was referred to the neurology department for conservative treatment. At 6 months follow up, the visual acuities improved to 0.2 in right eye and 0.3 in left eye.

### Case 2

A 72 year-old man presented with visual loss in his left eye for 7 months and decreased vision in his right eye for 8 months. He had been diagnosed with multiple lacunar cerebral infarctions and non-arteritic anterior ischemic optic neuropathy (NAAION) in the neurology department, but no positive neurological signs were found. He was given oral steroid therapy for several months, but with no improvement in vision. The patient had no history of hypertension or diabetes and no history of systemic or local infection.

**Fig. 2:**
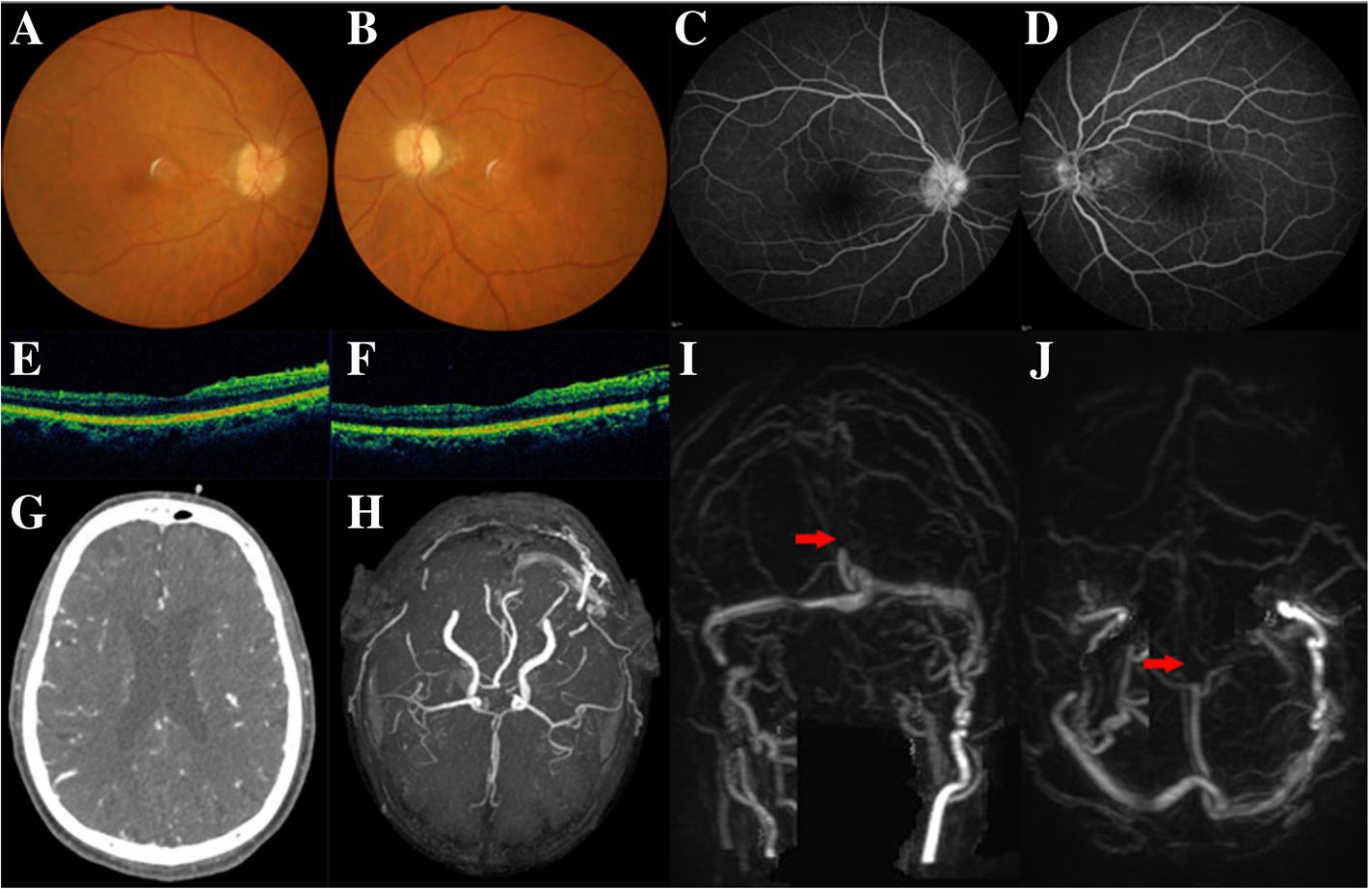
*Case 2, 72-year-old male patient with mild bilateral papilledema.* **a, b,** Ophthalmoscopy images. **c, d,** FFA images, showing hyperfluorescence of the right optic disc, and hypofluorescence in the left optic disc, without other abnormal leakage. **e, f,** OCT images, showing relatively normal macular morphology in both eyes. **g,** Contrast-enhanced CT image, showing no signs of elevated ICP. **h,** CTA image, showing a normal cerebral arterial system. **i**, **j,** MRV image, consistent with thrombosis (*red arrows*) of the superior sagittal sinus

The patient came to the ophthalmology outpatient department for further investigation. On presentation, he was in clear consciousness. Best corrected visual acuity was 0.3 (right) and no light perception (left). Relative afferent pupillary defect was present in the left eye. Mild lens opacity was observed in both eyes. In the right eye, the optic disc was slightly edematous (Fig. 2a). In the left eye, the optic disc was slightly pale in color (Fig. 2b). Signs as gliosis of peripapillary retinal nerve fiber layers, optociliary shunt vessels, or refractile bodies were not found. FFA showed hyperfluorescence of the right optic disc, and hypofluorescence in the left optic disc (Fig. 2c, d). HAP revealed superior and nasal scotomas (Fig. 4). OCT revealed that both macula had normal morphology (Fig. 2e, f). FVEP showed a mild decrease in amplitude of the P2 wave in the right eye, and a severe decrease in the left eye (Fig. 4). The ERG was relatively normal bilaterally. In the neurology department, he had previously undergone a contrast-enhanced CT-head (Fig. 2g) and CTA (computed tomographic angiography), which showed no abnormalities (Fig. 2h). An unenhanced MRI brain showed multiple lacunar cerebral infarctions and mild cerebral atrophy. Laboratory tests ruled out any blood disorders or infections. To further investigate for intracranial conditions, an MRV was performed, which demonstrated superior sagittal sinus thrombosis (Fig. 2i, j). LP showed an elevated cerebrospinal fluid (CSF) opening-pressure of 30 cm H_2_O. CSF protein, glucose, and cell counts were all within normal limits. The patient was referred back to the neurology department for endovascular intervention and stent placement. The best corrected visual acuity of right eye improved to 0.4 at six months following treatment.

### Case 3

A 20 year-old woman presented with a 1-month history of deteriorating vision. There was no history of eye pain, headache, vomiting, fever, or trauma. She denied any history of infection or surgery, and was not taking oral contraceptives, or any other medication. There was no notable past medical history.

**Fig. 3:**
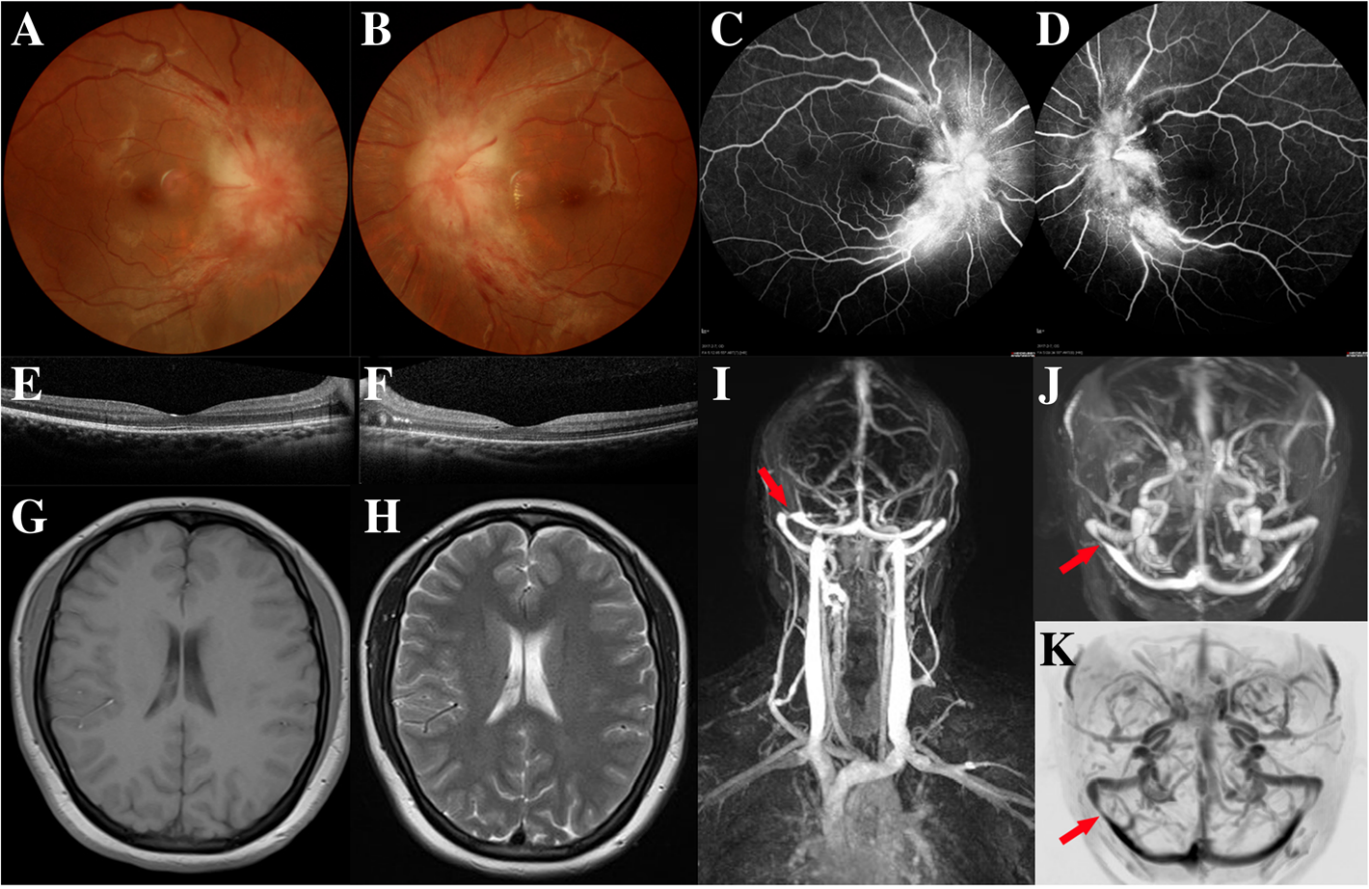
*Case 3, 20-year-old female patient with mild bilateral papilledema.* **a, b,** Ophthalmoscopy images. **c, d,** FFA images, showing hyperfluorescence of optic discs bilaterally, without other abnormal leakage. **e, f,** OCT images, showing normal macular morphology in both eyes. **g, h,** Unenhanced T1- and T2-weighted MRI images, respectively, showing no abnormalities. **i–k**, MRV images, showing a narrowed right transverse sinus (*red arrows*)

**Fig. 4:**
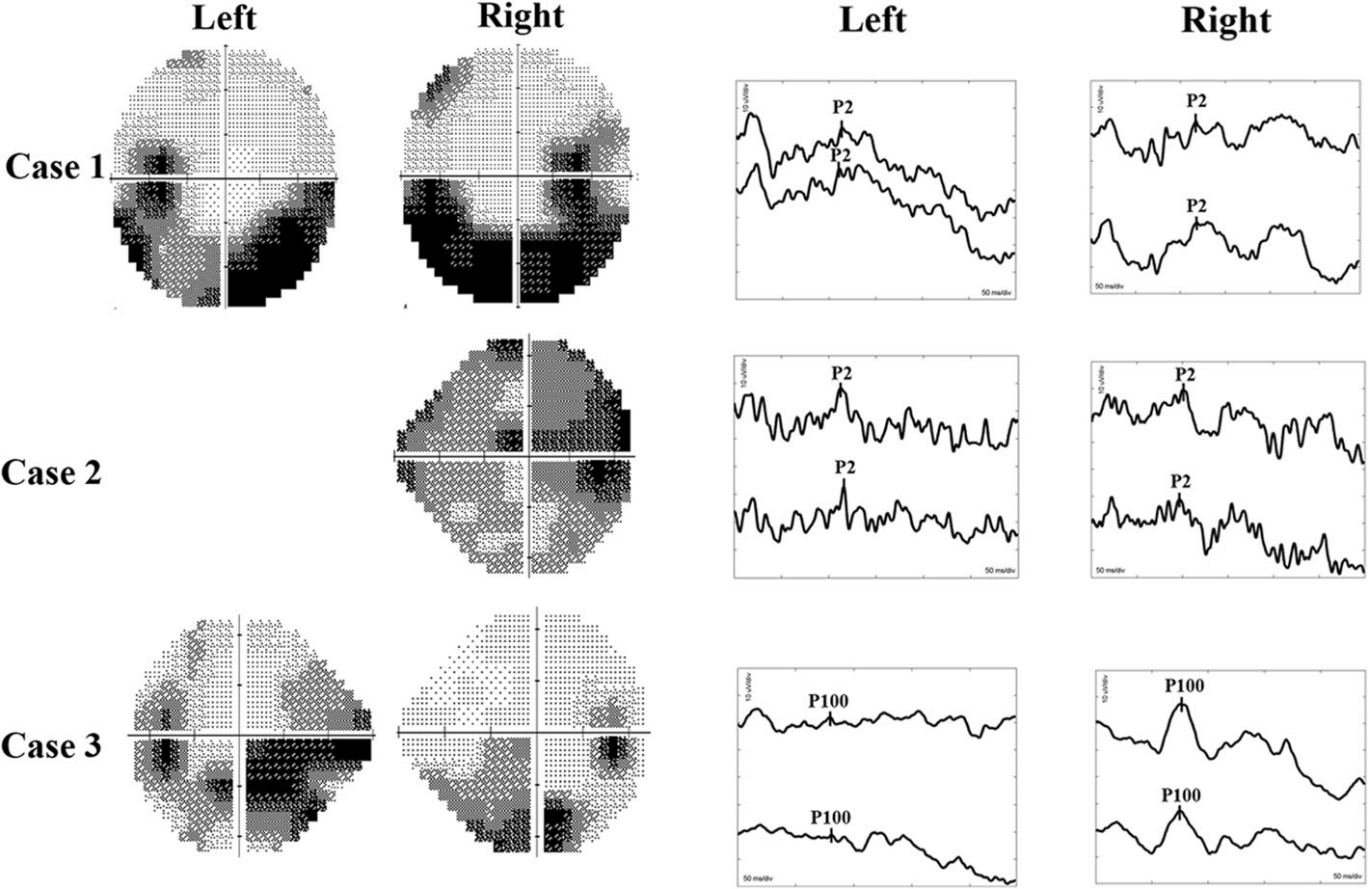
*Visual fields and FVEP/PVEP of the 3 cases*. Left, Humphrey visual field plots of both eyes. All patients had impairment of visual fields, secondary to papilledema. With case 2, the visual field of the left eye couldn’t be tested due to the poor vison. Right, FVEP plots for cases 1 and 2, showing only mild abnormalities (normal peak time, in contrast to delayed peak time in optic neuritis). PVEP plots for case 3, showing a nearly normal amplitude of the P100 wave in the right eye (visual acuity of 0.9) and a decreased amplitude with normal peak time of the P100 wave in the left eye (visual acuity of 0.5)

On presentation, the patient’s best corrected visual acuity was 0.9 (right) and 0.5 (left), and she had normal pupil diameter and pupillary reflexes. Ophthalmoscopy revealed significant bilateral optic disc swelling and peri-papillary hemorrhages, but no other abnormalities (Fig. 3a, b). The papilledema was surprisingly severe given her moderate visual impairment. The intra-ocular pressure (IOP) of both eyes was normal. Examined by neurologist, the patient showed no positive neurological signs. FFA showed hyperfluorescence of both optic discs and dilated peri-papillary capillaries (Fig. 3c, d). There was no other abnormal fluorescence observed. OCT showed bilateral papilledema but normal macular morphology (Fig. 3e, f). HAP showed non-specific bilateral inferior nasal scotomas (Fig. 4). Pattern VEPs (PVEPs) indicated a nearly normal amplitude of the P100 wave in the right eye (visual acuity of 0.9) and a decreased amplitude with normal peak time of the P100 wave in the left eye (Fig. 4).

Blood tests showed no evidence of systemic infection or biochemical abnormalities. No abnormalities were seen on an unenhanced MRI of the brain (Fig. 3g, h). An LP demonstrated an elevated CSF opening pressure of 29 cm H_2_O. To further investigate for intracranial conditions, an MRV was performed, which showed a narrowed right transverse sinus, but no other abnormalities (Fig. 3i–k). From this imaging, the patient was diagnosed with right transverse sinus stenosis and referred to the neurosurgery department for further investigation of venous anatomy, prior to treatment. At 6 months follow up, the visual acuity kept unchanged. Due to personal reasons, the patient refused the ventriculoperitoneal shunt suggested by neurologists.

## Discussion and conclusions

Papilledema is swelling of the optic nerve head (optic disc), secondary to elevated intracranial pressure (ICP) [4]. It is usually bilateral. Optic disc edema in the absence of elevated ICP is commonly referred to as “disc swelling”, which is usually associated with ocular diseases like optic neuritis and ischemic optic neuropathy. However, patients with bilateral optic disc swelling should be suspected of having an elevated ICP, whether or not they have neurological manifestations. Causes of elevated ICP include obstruction of the ventricular system by congenital or acquired lesions, space-occupying intracranial lesions, subarachnoid hemorrhage, cerebral trauma, cerebral venous sinus thrombosis/stenosis, and idiopathic intracranial hypertension.

Elevated ICP usually causes dramatic neurological symptoms, including headaches, nausea, vomiting and deterioration of consciousness, as well as visual impairment. Such patients are most commonly diagnosed in the neurology department. However, chronic cerebral venous sinus occlusion (CVSO), either complete or partial, can cause chronically elevated ICP, and can produce visual impairment as the only clinical symptom. Thus such patients often present to the optometrist or ophthalmologist. As well as producing decreased visual acuity, chronic papilledema induced by elevated ICP can produce visual field defects if the optic disc edema persists. However, unlike primary optic nerve diseases like optic neuritis, the flash VEP in patients with papilledema secondary to elevated ICP is often normal or only mildly affected [5, 6].

In case 1, the symptoms, other than blurred vison, were relatively occult. Although the bilateral optic disc swelling strongly indicated elevated ICP, there were no positive findings on CT or MRI. However, vision was not improved following treatment with systemic steroids. Therefore, further investigation was performed, which demonstrated elevated ICP secondary to right sigmoid sinus thrombosis. In retrospect, the patient’s history of mastoiditis and surgery eight years prior was highly significant. Mastoiditis is an important cause of sigmoid sinus thrombosis, especially in younger patients.

In case 2, the patient was a septuagenarian male with a long history of impaired vision, but no neurological symptoms. Due to the likely chronicity of the elevated ICP at presentation, the optic disc swelling in the right eye was not prominent, and the optic nerve in the left eye was already slightly atrophied, and the eye was blind. Prior to ophthalmology consultation, his visual impairment may be partially attributed to his multiple lacunar cerebral infarctions. But this multiple lacunar cerebral infarction as Fig. 3 showed can’t explain the severity of his visual impairment of both eyes, which indicated that the cerebral infarctions did not distinctly affect his visual pathway. Given his age, a diagnosis of non-arteritic anterior ischemic optic neuropathy was also made. However, the severity of visual impairment and lack of response to steroid therapy indicated the need for further investigation. Following MRV imaging, a diagnosis of superior sagittal sinus thrombosis was made. The patient’s visual impairment improved following endovascular stenting of the venous sinus.

Case 3 was that of a young adult female with blurred vision and bilateral papilledema, but no other neurological symptoms, and no history of systemic disease or infection. Her elevated ICP was confirmed by lumbar puncture, but unlike the other two cases, her radiological findings were less prominent. There were no definite signs of sinus thrombosis observed from the MRV, but a narrowed section was noted at the junction between the right transverse and sigmoid sinus. With no evidence of any intracranial mass, the patient was diagnosed with right transverse sinus stenosis. However, the etiology of this stenosis is currently still unknown, and the patient is under continued management by the neurosurgical team.

CVSO (partial or complete) affects the dural venous sinuses that drain blood from the brain, and is usually caused by either venous thrombosis or stenosis. Cerebral venous sinus thrombosis (CVST) most commonly affects the transverse sinus (86% of cases), followed by the superior sagittal sinus (62%), straight sinus (18%), then least commonly, the cortical veins (17%) [1]. Risk factors for CVST include thrombophilia, chronic inflammatory diseases, use of hormonal contraception, infections such as meningitis, mastoiditis and sinusitis, and invasive procedures in the head and neck area [7, 8]. Symptoms of CVST include headache, visual impairment, symptoms of stroke (such as unilateral limb and facial weakness), and seizures. However, neurological symptoms are absent in a notable proportion of patients, and these patients may present later with impaired visual acuity due to chronic, advancing papilledema [9, 10].

Cerebral venous sinus stenosis (CVSS) is a rare intracranial abnormality. Stenosis may be caused by abnormal intrinsic dural sinus anatomy or by extrinsic compression; for example, due to an intracranial tumor or enlarged arachnoid granulation. In many cases the cause of stenosis is unknown. The stenosis is most often found at the junction of the transverse and sigmoid sinuses, and is typically diagnosed by venography [11, 12]. Regardless of the underlying cause, stenting has proved (in multiple retrospective, non-controlled studies) to be an effective method for improving the symptoms of elevated ICP and papilledema [13, 14].

Imaging of patients with CVSO, using unenhanced CT or MRI, may demonstrate gross abnormalities, such as cerebral edema, venous infarction and dilated ventricles. However in some cases, unenhanced imaging may be totally normal, especially in patients with a chronic disease course. Therefore, when elevated ICP is suspected, contrast-enhanced MR venography is required to image the cerebral veins for thrombus or stenosis, and lumbar puncture (LP) may be needed, to measure the opening pressure and test for constituent changes in the CSF.

In summary, CVST and CVSS (here collectively referred to as CVSO) are severe conditions which can cause elevated ICP, leading to visual impairment. A considerable amount of patients with CVSO have no apparent neurological symptoms other than visual loss. VEP could be normal or abnormal. When encountering patients with bilateral papilledema, especially those with normal unenhanced brain imaging, CVSO should always be considered. Further investigations such as LP, MRV or DSA are necessary to diagnose or rule-out CVST or CVSS.

## Abbreviations

CSF: Cerebrospinal fluid
CTA: Computed tomographic angiography
CVSO: Cerebral venous sinus occlusion
CVSS: Cerebral venous sinus stenosis
CVST: Cerebral venous sinus thrombosis
DSA: Digital subtraction angiography
ERG: Electroretinogram
FFA: Fundus fluorescein angiography
HAP: Humphrey automated perimetry
ICP: Intracranial pressure
IOP: Intra-ocular pressure
LP: Lumbar puncture
MRI: Magnetic resonance imaging
MRV: Magnetic resonance venography
NAAION: Non-arteritic anterior ischemic optic neuropathy
OCT: Optical coherence tomography
VEPs: Visual evoked potentials
